# Reduced blood pressure in sickle cell disease is associated with decreased angiotensin converting enzyme (ACE) activity and is not modulated by ACE inhibition

**DOI:** 10.1371/journal.pone.0263424

**Published:** 2022-02-03

**Authors:** Pamela L. Brito, Alisson F. dos Santos, Hanan Chweih, Maria E. Favero, Erica M. F. Gotardo, Juliete A. F. Silva, Flavia C. Leonardo, Carla F. Franco-Penteado, Mariana G. de Oliveira, Wilson A. Ferreira, Bruna C. Zaidan, Athanase Billis, Giorgio Baldanzi, Denise A. Mashima, Edson Antunes, Sara T. Olalla Saad, Fernando F. Costa, Nicola Conran

**Affiliations:** 1 Hematology and Transfusion Center, University of Campinas-UNICAMP, Campinas, São Paulo, Brazil; 2 Centro de Hematologia e Hemoterapia do Paraná, Curitiba, Paraná, Brazil; 3 University Hospital, State University of Londrina, Londrina, Brazil; 4 Department of Pharmacology, University of Campinas-UNICAMP, Campinas, São Paulo, Brazil; 5 Department of Pathology, School of Medical Sciences, University of Campinas-UNICAMP, Campinas, São Paulo, Brazil; Université Claude Bernard Lyon 1, FRANCE

## Abstract

**Background:**

Sickle cell disease (SCD) incurs vaso-occlusive episodes and organ damage, including nephropathy. Despite displaying characteristics of vascular dysfunction, SCD patients tend to present relatively lower systemic blood pressure (BP), via an unknown mechanism. We investigated associations between BP and renin-angiotensin-system (RAS) components in SCD and determined whether an inhibitor of angiotensin converting enzyme (ACE; often used to slow SCD glomerulopathy) further modulates BP and RAS components in a murine model of SCD.

**Methods:**

BP was compared in human subjects and mice with/without SCD. Plasma angiotensin II, ACE and renin were measured by immunoassay. BP was reevaluated after treating mice with enalapril (25 mg/kg, 5x/week) for 5 weeks; plasma and organs were stored for angiotensin II and ACE activity measurement, and quantitative real-time PCR.

**Results:**

Diastolic BP and systolic BP were significantly lower in patients and mice with SCD, respectively, compared to controls. Reduced BP was associated with increased plasma renin and markers of kidney damage (mice) in SCD, as well as significantly decreased plasma ACE concentrations and ACE enzyme activity. As expected, enalapril administration lowered BP, plasma angiotensin II and organ ACE activity in control mice. In contrast, enalapril did not further reduce BP or organ ACE activity in SCD mice; however, plasma angiotensin II and renin levels were found to be significantly higher in enalapril-treated SCD mice than those of treated control mice.

**Conclusion:**

Relative hypotension was confirmed in a murine model of SCD, in association with decreased ACE concentrations in both human and murine disease. Given that ACE inhibition has an accepted role in decreasing BP, further studies should investigate mechanisms by which ACE depletion, via both Ang II-dependent and alternative pathways, could contribute to reduce BP in SCD and understand how ACE inhibition confers Ang II-independent benefits on kidney function in SCD.

## Introduction

Sickle cell disease (SCD) is an inherited hemoglobinopathy that arises from the synthesis of abnormal hemoglobin (Hb) S. HbS polymerizes under deoxygenating conditions, resulting in the sickling of red blood cells and multiple pathophysiological consequences that can result in reduced life expectancy and acute and chronic complications, including characteristic frequent painful vaso-occlusive episodes that often require hospitalization [1].

Despite significant alterations in oxidative stress, nitric oxide signaling, the endothelin system, and eicosanoid levels in SCD [2–5], all of which incur cardiovascular adaptations, numerous studies have reported a tendency for individuals with SCD to display lower systemic blood pressure (BP), when compared with age- sex- and race-matched controls [6–9]. Additionally, significant positive correlations of systemic BP (SBP) and diastolic BP (DBP) with age and body mass index have been described in cohorts of SCD individuals [9, 10], as well as a negative correlation between SBP and absolute neutrophil count.

The mechanism leading to reduced BP in SCD, however, is unclear, and while exacerbated urinary sodium loss due to hyposthenuria has been suggested to contribute to relative hypotension in SCD [9], evidence for this hypothesis is lacking [11]. The renin-angiotensin system (RAS), a vasoactive hormonal system, is a major regulator of BP and fluid balance that responds to hemodynamic instability, in order to avoid reductions in systemic tissue perfusion [12]. The RAS consists of a conventional vasopressor pathway whereby renin, a proteolytic enzyme that is synthesized, stored and secreted by the cells of the juxtaglomerular apparatus in response to reduced afferent arteriolar pressure, cleaves angiotensinogen, produced by the liver, to form angiotensin I (Ang I). In turn, angiotensin converting enzyme (ACE; an ectoenzyme), found primarily on the surface of endothelial cells, cleaves Ang I to the 8 amino acid peptide, Angiotensin II (Ang II), which has potent short-term vasoconstrictor properties. ACE also has other effects that are independent of Ang II generation, including bradykinin hydrolysis [13, 14]. On the other hand, ACE-2, a carboxypeptidase that displays homology to ACE, exerts protective effects, converting Ang II to Ang (1–7), a vasodilator peptide [14]. ACE-2 also catalyzes the production of Ang (1–9) from Ang I, which can then be cleaved to form Ang (1–7). The biological effects of Ang II are mediated by two types of receptors, the angiotensin II receptor type 1 (AT1R) and angiotensin II receptor type 2 (AT2R). The binding of Ang II to AT1Rs results in vasoconstriction, inflammation and sodium reabsorption effects, while the binding of Ang II to AT2Rs may favor vasodilation [15, 16].

Kidney damage is a frequent complication of SCD, where glomerular and tubular disorders are associated with renal failure and increased mortality in these patients [17–19]. Red cell sickling in the renal medulla has been suggested to result in continuous vaso-occlusive processes in the kidney and it is possible that renal ischemia, leading to the increase in glomerular filtration rate that is often observed in SCD [19], may result in reduced renal blood flow and alterations in the RAS. Indeed, inhibitors of ACE (ACEi) have been employed with a view to preventing or reducing kidney complications or chronic kidney disease in people with SCD [20]. In a previous study conducted by our research group, decreased expression of the gene encoding ACE in the kidneys of mice with SCD was observed, in association with decreased plasma levels of Ang II in the plasma of these mice [21].

Thus, this study aimed to investigate possible associations between BP reduction and changes in proteins of the RAS and kidney function in patients with homozygous sickle cell anemia (SCA) and in an animal model of SCD. Additionally, given that ACEi are sometimes used in SCD with a view to preventing the progression of glomerulopathy [22], we investigated the effects of an inhibitor of ACE, enalapril, on the BP and RAS components of mice with SCD, to observe the potential hypotensive effects of such drugs and further understand the mechanism of action of these approaches.

## Materials and methods

### Human subjects

A total of 58 male and female patients with homozygous SCA, aged 13–51 years, participated in the study. Patients, diagnosed by hemoglobin electrophoresis and high pressure liquid chromatography (HPLC) methods, were treated at the Hematology Center, University of Campinas, the Hematology Center of Paraná (HEMEPAR) and at the University Hospital, State University of Londrina. Patients were in steady state and had not presented with vaso-occlusive episodes nor received blood transfusion during the 3 months prior to participation in the study. None of the patients were in use of ACE inhibitors, or presented a diagnosis of renal disease. Those patients on hydroxyurea therapy (N = 28, 5–30 mg / kg / day) had been taking it for at least two months. Healthy volunteers (total of 40) were age- and sex-matched where possible and did not use antihypertensive drugs. All individuals provided written informed consent and the study was approved by the Research Ethics Committee of the Faculty of Medical Sciences of UNICAMP (CAAE: 0898.0.146.000–09) and by the Research Ethics Committee of the State University of Londrina—UEL (n° 3113/2011).

### Animals

Male control chimeric mice (CON) and sickle cell disease mice (SCD) were obtained by the transplantation of bone marrow from C57BL6 / JuniB or transgenic mice with SCD (Berkeley strain; Hbatm1Paz Hbbtm1Tow Tg (HBA-HBBs) 41Paz / J) [23] to 8-week-old irradiated C57BL6 / JuniB mice, respectively, as previously described. Mice were obtained from the animal house (CEMIB), at the University of Campinas. Animals were phenotyped to confirm graft success at 2 months post-transplant, according to [21], and were then used at the ages of 22 weeks (5 months; “young adults") and at 37 weeks (9 months; adults), according to [24]. Male mice were chosen for use in animal protocols due to the potential for modulation of the RAS by hormonal variations in females [25].

The mice were housed five animals/ cage with food and water ad libitum, and controlled light and temperature conditions. All animal experimental procedures were carried out in accordance with the ‘Principles of Laboratory animal care’ (http://grants.nih.gov/grants/guide/notice-files/not96-208.html), as well as in accordance with current Brazilian laws for the protection of animals. The study was approved by the Ethics Committee for the Use of Animals, University of Campinas (UNICAMP; protocols 4568–1 / 2017 and 4568–1 / 2017 (A)). All efforts were made to minimize suffering, animals were monitored daily and no unexpected adverse events occurred. All animals were included in each endpoint analysis and animal numbers are included in the figure caption descriptions. For blood collection, animals were anesthetized (80mg/kg ketamine; 10mg /kg xylazine, i.p.) and, at the end of the experiments, the mice were euthanatized using a lethal dose of ketamine (300mg/kg) and xylazine (30mg/kg, i.p.), followed by cervical dislocation.

### Animal treatments and blood collection

For enalapril treatment, chimeric mice (5-months old) received enalapril (25 mg/kg, gavage) for 5 days/week for 5 weeks. Untreated mice groups were not treated with enalapril or received drug vehicle (water) administration alone during the same period; drug vehicle and non-treatment yielded statistically similar data for all parameters measured. Blood pressure was measured during the last week of enalapril administration and mice were euthanized 72 h after administration of the final enalapril dose. Peripheral blood was collected from human subjects in EDTA or heparin for Ang II and ACE measurement, respectively. After collection, a protease inhibitor cocktail (Angiotensin II Inhibitor Cocktail–Cayman Chemical, Ann Arbor, Michigan, USA) was added immediately to the tube to avoid Ang II degradation, according to the manufacturer’s instructions. For mice, blood samples were collected in EDTA and Angiotensin II Inhibitor Cocktail added, as before. After centrifugation of blood samples at 3000 *g* (4°C for 15 min), plasmas were separated and immediately stored at -80°C until assay.

### Measurement of plasma angiotensin II

Ang II was extracted from plasma samples (containing Ang II inhibitor cocktail) using phenyl Hypersep PH columns (Thermo Scientific, Waltham, MA, USA) before determining Ang II concentrations using the Angiotensin II Enzyme Immunoassay (Cayman Chemical, Ann Arbor, Michigan, USA), according to the manufacturer’s instructions. Final plasma Ang II concentrations were calculated from a standard curve and adjusted according to the initial plasma volume from which Ang II was extracted.

### Measurement of plasma ACE and renin

Plasma ACE was measured in duplicate heparin-anticoagulated human plasma samples using the Quantikine Human ACE ELISA (R&D Systems, Minneapolis, MN, USA), according to the manufacturer’s instructions. Plasma ACE and renin were quantified in duplicate mouse plasma samples using the Mouse ACE and Mouse Renin 1 ELISAs from Abcam, respectively (Cambridge, UK). The presence of the Ang II cocktail inhibitor in mice plasma samples was found not to alter the detection of ACE or renin by these kits.

### Monitoring of blood pressure in humans and mice

Systolic and diastolic BPs were monitored in human subjects in the fed state during routine visits, in the sitting position, and using a mercury sphygmomanometer. Systolic BP was determined in mice by the indirect non-invasive measurement of the tail blood pressure using a mouse tail cuff transducer and an integrated equipment and software system (PowerLab and LabChart 7—AdInstruments, Colorado Springs, CO, USA). For measurements, mice tails were previously heated (37°C) for 8 min to permit vasodilation, and each animal was placed individually in a mouse container (Rodent Restrainer MLA5018—AdInstruments) for tail cuff pressure measurement [26]. All animals were habituated to the blood pressure procedure for two weeks (thrice weekly) prior to the experimental period and systolic BP was calculated as the mean of pulsatile data collected during three measurements/day on three different days in the space of one week during the experimental period (LabChart7 –AdInstruments). Systolic BP measurements, made using non-invasive tail cuff measurements in warmed and restrained WT mice, have previously been shown to strongly correlate with arterial blood pressure measurements made using an implanted radiotelemetry pressure transducer [27].

### Immunohistochemistry

Kidneys were collected from mice, at the time of sacrifice, and immersed in OCT mounting medium (Tissue Tek^TM^; Thermo Fischer Scientific, Waltham, MA, USA). Serial cryostat sections (M185 Leica Cryostat) of 10-μm thickness were then obtained and placed on silanized slides before storage at -80°C. For immunohistochemistry, slides were thawed at 37°C for 20 minutes and the tissues fixed with 4% paraformaldehyde for 15 minutes at room temperature. After successive washes with PBS and 0.1% Tween 20 (PBST), the sections were treated with methanol solution and 30 v/v hydrogen peroxide (1:1) for tissue autofluorescence blocking. Subsequently, nonspecific binding sites were blocked with 3% BSA solution during 1 h at room temperature, after which the specimens were incubated overnight at 4°C with rabbit anti-ACE and goat anti-angiotensin (1:100 dilution, Santa Cruz Biotechnology Inc., Dallas, TX, USA). The secondary antibodies, donkey anti-Goat IgG Alexa Fluor® 488 conjugate and donkey anti-Rabbit IgG Alexa Fluor® 555 conjugate (1: 1000, Santa Cruz Biotechnology, Inc.) were then applied along with DAPI (4 ’, 6-Diamidino-2-phenylindole; Sigma Aldrich, San Luis, Missouri, USA) for labeling the cell nuclei, for 1 h at room temperature. Slides were visualized using a fluorescence microscope (Axio Imager D.2, Carl Zeiss, Jena, Germany), and images captured using an Axiocam 506 Color (Carl Zeiss) and processed using Zeiss Zen Imaging software (Carl Zeiss).

### ACE assay activity measurement

ACE activity was determined in heparin-anticoagulated human plasma samples and murine organ extracts. Organs were dissected from mice at the time of euthanasia and snap-frozen in liquid nitrogen and stored at -80° C until the day of the assay. Tissue homogenates were obtained as described by [28], with modifications. To obtain the total extract, the tissues were homogenized in extraction buffer (50 mM Hepes, pH 7.4, 150 mM NaCl, 0.5% Triton X-100, 25 mM ZnCl_2_, 1 mM phenylmethanesulfonyl fluoride), and the homogenate obtained was centrifuged at 10 000 *g* for 15 min at 4°C. The supernatant was collected and centrifuged again at 10 000 *g* for 10 min at 4°C. Immediately after extraction, total protein dosage was performed using the Bradford assay [29]. ACE activity was measured using the commercial Angiotensin I Converting Enzyme (ACE) Activity Assay kit (Sigma Aldrich), following the manufacturer’s instructions. The kinetic reaction of the enzyme was evaluated using the SpectraMax i3X fluorescence reader (Molecular Devices, San José, CA, USA) with excitation at 320 nm and emission at 405nm. To obtain the enzyme activity values, a standard curve (RFU / nmol) was used and the values of the samples were corrected by the total protein content.

### Quantitative real time-PCR (qPCR)

Organs were dissected from mice at the time of euthanasia, snap-frozen in liquid nitrogen and stored at -80°C until the day of assay. mRNA was extracted from entire organs using Trizol (Invitrogen, Carlsbad, CA, USA) and cDNA was synthesized using a reverse transcription kit (RevertAid H Minus First Strand cDNA Synthesis, Thermo Scientific, Waltham, MA, USA). Synthetic oligonucleotide primers were designed (Primer-Express; Applied Biosystems, Foster City, CA, USA) to amplify cDNA for conserved regions of the genes encoding ACE (*Ace*), ACE-2 (*Ace2*), KIM-1 (Kidney injury molecule-1; *Havcr1*), MMP9 (matrix-metalloproteinase 9; *Mmp9*), MCP-4 (murine mast cell protease 4; *Mcpt4*), and LCN2 (or neutrophil gelatinase-associated lipocalin, NGAL; *Lcn2*) (for primer sequences, see S1 Table). Primers were synthesized by IDT (Coralville, Iowa, USA). All samples were assayed in a 12 μL volume containing 10 ng cDNA, 6 μL SYBR Green Master Mix PCR (Applied Biosystems) and gene primers, using a 7500 Fast Real-Time PCR System (Applied Biosystems). To confirm the accuracy and reproducibility of the real-time PCR, the intra-assay precision was calculated according to the equation: E(−1 / slope). The dissociation protocol was performed at the end of each run to check for non-specific amplification. Two replicas were run on the plate for each sample. Results are expressed as arbitrary units (A.U.) of gene expression, normalized according to the expressions of Actb and Gpdh, performed with the geNorm program [30].

### Statistical analysis

Data are expressed as means and standard error of N samples. For comparisons between two groups, the non-parametric Mann-Whitney test was used. For comparisons between three groups or more, the One-Way Analysis of Variance (ANOVA) test was applied followed by Dunn’s multiple-comparison test (non-parametric samples) or Bonferroni (parametric samples). Spearman’s test was used for the correlation of non-parametric samples. A p value of less than or equal to 0.05 (p ≤0.05) was considered statistically significant.

## Results

### Blood pressure is decreased in patients and mice with SCA

**Fig 1:**
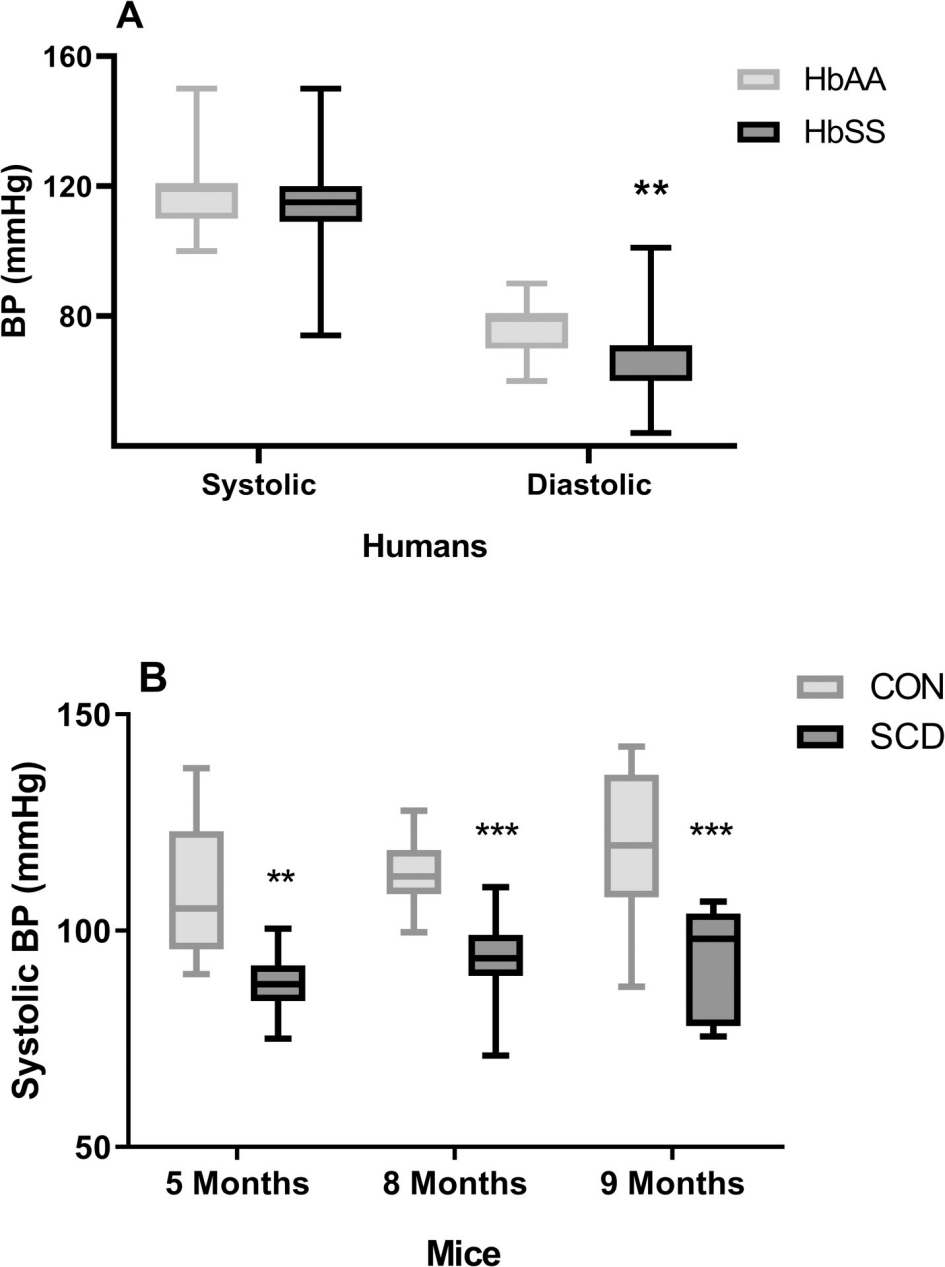
Blood pressure is reduced in human and murine sickle cell disease. (A) Systolic and diastolic blood pressures were determined in age-matched healthy control (HbAA, N = 35) individuals and patients with SCA (HbSS, N = 58). (B) Mean systolic arterial blood pressure (BP) was determined in SCD (SCD, N = 6–12) and control (CON, N = 10–14) chimeric mice at 5, 8 and 9 months of age, data are means of three measurements on three different days. **, P<0.01; ***, P<0.001, compared to HbAA or CON.

We compared systolic and diastolic BP in patients with SCA (HbSS), in steady state, to those of healthy aged-matched individuals without SCA (HbAA) (HbAA, 28.1±1.5 yrs; HbSS, 28.2±1.3 yrs, N = 40 and 58, respectively; p>0.05). As previously observed, diastolic BP was found to be slightly decreased in SCA patients, compared to healthy controls (Fig 1A; HbAA, 74.9±1.5 mmHg; HbSS, 68.1±1.4 mmHg, P<0.05). The relatively lower systolic BP measured in individuals with SCA was not significantly lower than that of the systolic BP in healthy controls (Fig 1A; HbAA, 118.5±2.0 mmHg; HbSS, 114.7±1.7 mmHg, P>0.05). In association with this finding, mean systolic arterial BP was found to be significantly decreased in chimeric SCD mice (P<0.01), when compared with chimeric control (CON) mice at 5, 8 and 9 months of age (young adult and adult phases) (Fig 1B).

No significant differences were observed in BP in SCA patients when these were stratified according to use, or not, of hydroxyurea therapy (the standard of care for SCA at our center; S1A Fig) or when stratified according to age (S2A and S2B Fig). Correlation analysis of BP in SCA with hematological parameters revealed no significant associations (S2 Table).

### Evidence for impaired kidney function or damage in the human and murine SCA subjects studied

Urine microalbuminuria and glomerular filtration rates were not available for most of the patients included in this study. However, both serum urea and creatinine concentrations were found to be significantly lower in our SCA patient population, compared to control individuals (Serum urea: 30.20 ± 1.01 and 20.73± 1.11 mg/dL; serum creatinine; 0.80 ± 0.03 and 0.57 ± 0.02 mg/dL for control (N = 30) and SCA (N = 52) individuals, respectively. P< 0.0001). While no significant association between RAS protein concentrations and BP were observed in the SCA patient population, we did find a negative correlation between systolic BP and serum urea (P<0.05; S3 Table).

Comparison of the expressions of *Lcn2* (the gene encoding the NGAL biomarker), *Havcr1* (encoding KIM-1) and Mmp9 (encoding MMP9), by qPCR, in the kidneys of CON and SCD mice indicated the expressions of *Lcn2* and *Mmp9* to be significantly elevated in the SCD mice, indicating the existence of kidney damage (Fig 5G–5I).

### Circulating RAS protein levels are modulated in SCA

**Fig 2:**
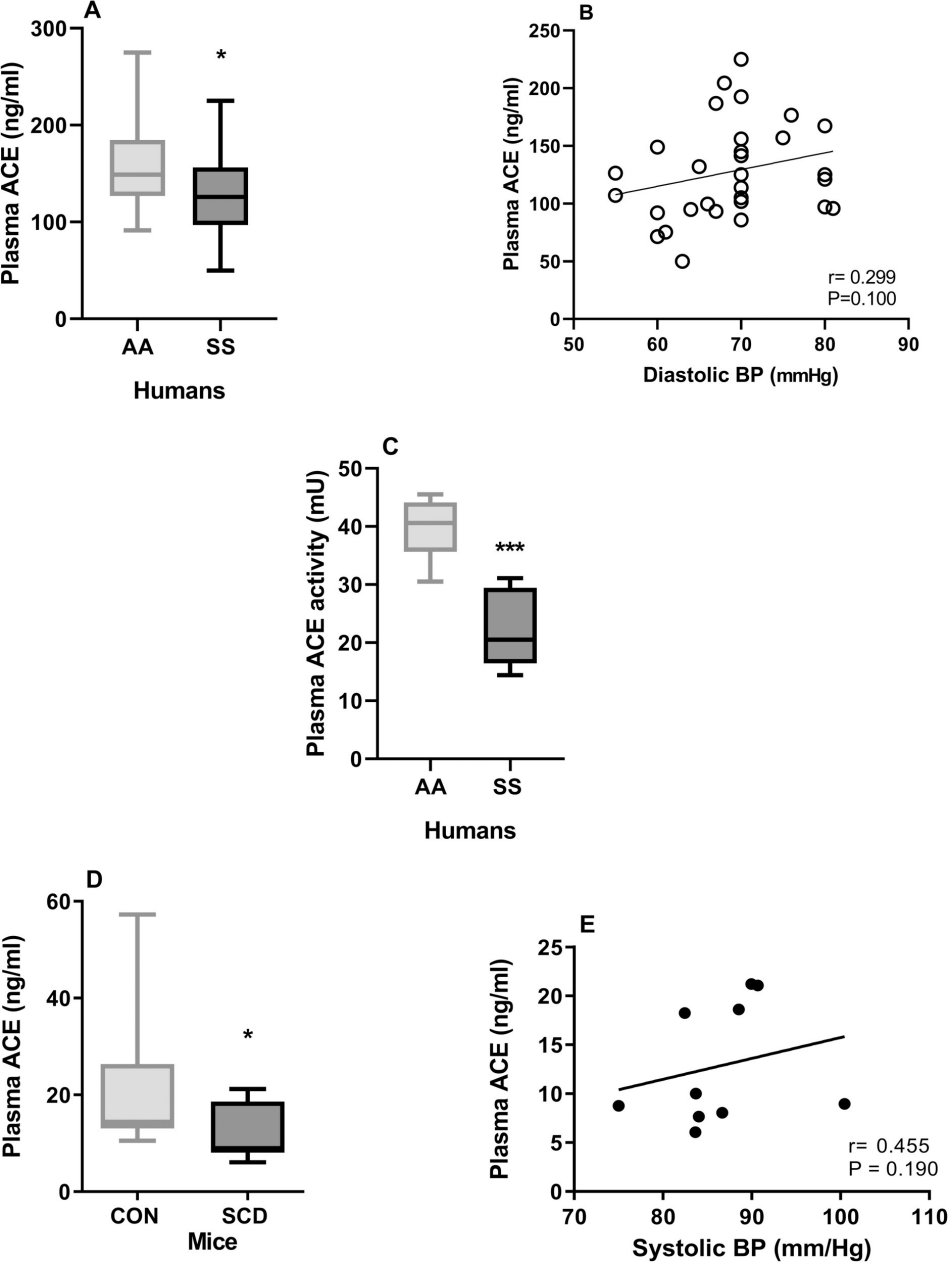
Measurement of plasma ACE in SCD. (A) Plasma ACE in patients with SCA (SS, N = 34) and healthy individuals (AA, N = 29). (B) Correlation between diastolic BP and ACE in SCA patients (N = 31). (C) Activity of ACE in the plasma of patients with SCA (SS, N = 12) and healthy individuals (AA, N = 6). (D) Plasma ACE in chimeric mice with SCD (SCD, N = 11) and control (CON, N = 9) mice at 5 months of age. (E) Correlation between mean systolic BP and ACE in SCD mice. Plasma ACE was measured by ELISA. *, P<0.05; **, P<0.01; ***, P<0.001 compared to AA or CON.

Given the importance of the RAS in regulating BP, and the alterations indicative of kidney function impairment observed, we measured plasma levels RAS components in patients with steady state SCA and in chimeric SCD mice and compared these with levels in healthy control individuals and in chimeric CON mice. Concentrations of plasma ACE, responsible for Ang II cleavage from angiotensin I, were significantly decreased in both patients with SCA and in young adult mice with SCD, when compared with their respective controls (Fig 2A and 2D). Furthermore, plasma ACE activity was significantly lower in patients with SCA, compared to healthy control individuals (Fig 2C). Positive correlations of plasma ACE concentrations with diastolic BP were observed in patients with SCA (Fig 2B) and BP in SCD Mice (Fig 2E), but these were not found to be significant. Hydroxyurea therapy was not associated with any modulation of plasma ACE in the SCA patient population (S1B Fig). A tendency towards a decrease in plasma ACE II occurred in adult SCA patients, compared to teenage patients (S2C Fig), and this trend was significant when comparing ACE concentrations in patients of 20–29 years with those of less than 20 years (S2D Fig). No correlations between plasma ACE concentrations and hematological findings were observed in the SCA patient population (S2 Table).

**Fig 3:**
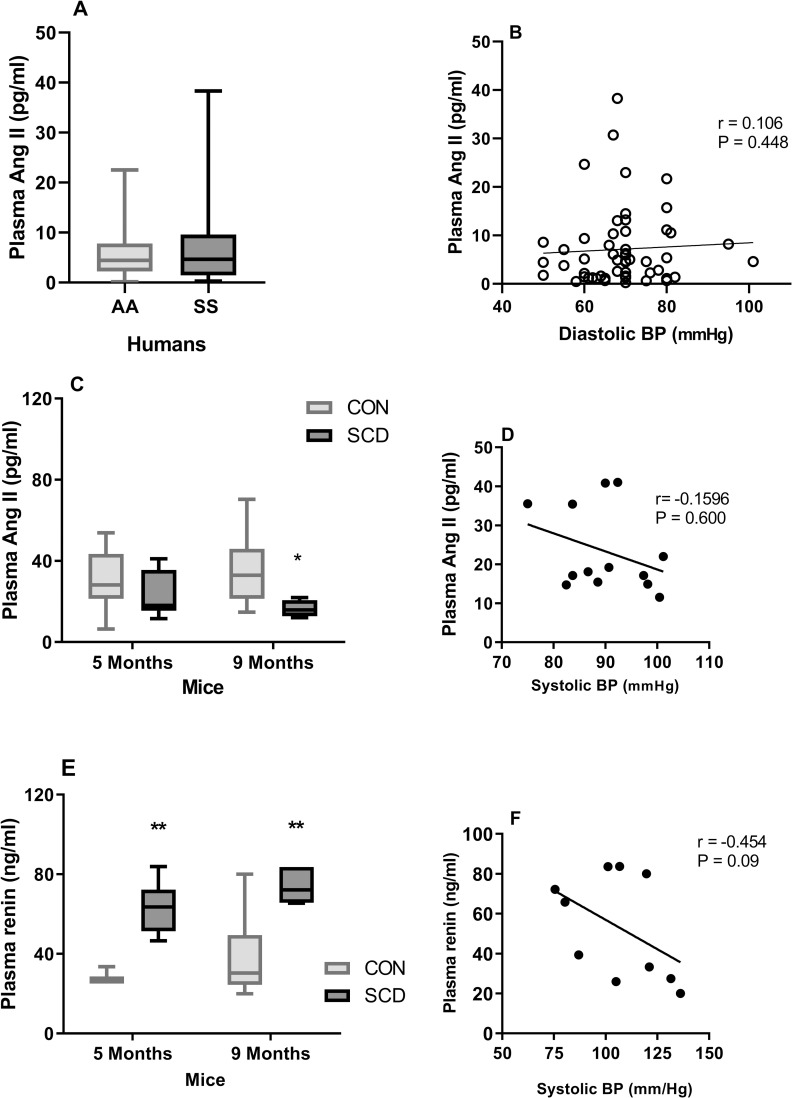
Measurement of plasma Ang II and renin in SCD. (A) Plasma Ang II in patients with SCA (SS, N = 58) and healthy individuals (AA, N = 40). (B) Correlation between diastolic BP and Ang II in SCA patients (N = 54). (C) Plasma Ang II in chimeric mice with SCD (SCD) and control (CON) mice at 5 and 9 months of age (N = 4–11). (D) Correlation between mean systolic BP and Ang II in SCD mice. (E) Plasma renin in chimeric mice with SCD (SCD) and control (CON) mice at 5 and 9 months of age (N = 3–6). (F) Correlation between mean systolic BP and renin in SCD mice. Plasma Ang II and renin were measured by ELISA. *, P<0.05; **, P<0.01, compared to CON.

Measurement of plasma Ang II, the principal pressor effector of the RAS, by ELISA demonstrated no significant alteration in the levels of this protein between healthy control (HbAA) and SCA (HbSS) individuals (Fig 3A). Accordingly, no significant differences were observed in plasma Ang II concentrations in SCA patients when these were stratified according to use of hydroxyurea therapy, or not, or according to age (S1C Fig, S2E and S2F Fig). We also found no significant change in Ang II plasma concentrations in young adult SCD mice, but we did observe a reduction in plasma Ang II in adult SCD mice, when compared with age-matched CON mice (Fig 3C), as previously reported [21]. In mice with SCD, ACE correlated very significantly with Ang II (r^s^ = 0.996, P<0.0001, n = 9), although this correlation was not observed in SCA patients (S3 Table). Levels of Ang II did not correlate significantly with BP in either the human or murine SCA groups (Fig 3B and 3D) and did not correlate with hematological data in the SCA patient group (S2 Table).

In contrast, plasma levels of the protease, renin, were significantly increased in young adult and adult mice with SCD, when compared with control mice of the same age (Fig 3E). Despite a trend towards a negative correlation between plasma renin and BP in SCD, this correlation was not significant (Fig 3F).

**Fig 4:**
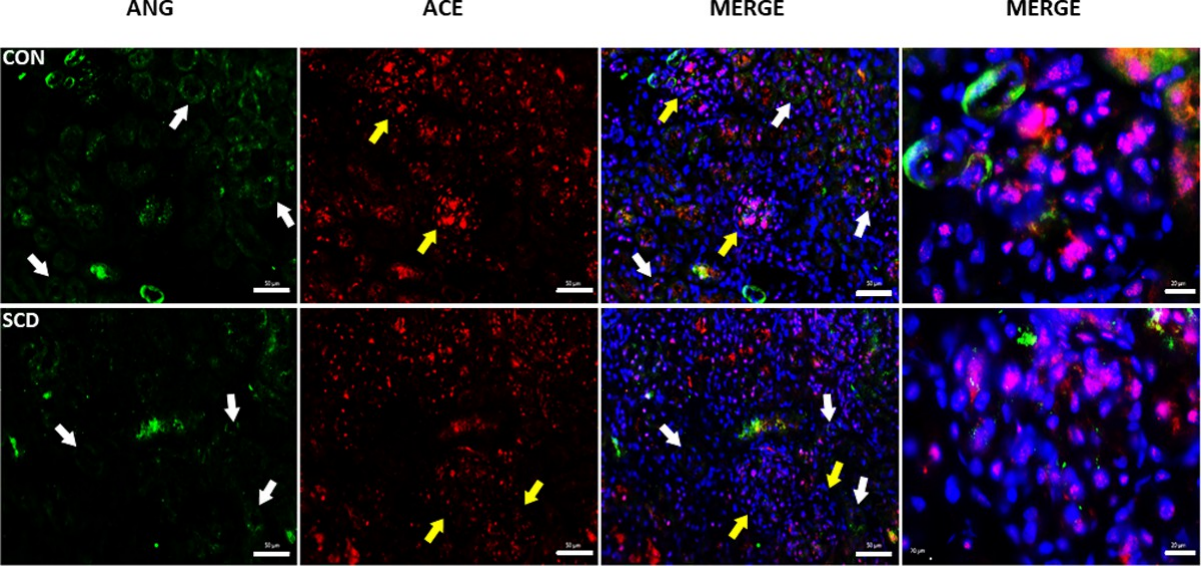
Expression and distribution of angiotensin and ACE in the kidneys of control (CON) and SCD (SCD) mice. Immunofluorescent staining of kidney sections for angiotensin and ACE. Angiotensin (I, II and III) was stained with anti-angiotensin and AlexaFluor®488-conjugated secondary antibody (green); ACE was detected with anti-ACE and AlexaFluor®555-conjugated secondary antibody (red); nuclei were stained with DAPI (blue). The arrows show tubule (white) and glomerular (yellow) structures. Images are representative of three kidney sections from N = 3 mice. Scale bar = 50 μm and 20 μm (Magnified merge).

Immunohistochemical staining of sections of kidneys from CON and SCD mice at 5 months of age demonstrated no significant alterations in the expression of angiotensin (I, II and III) proximal to the tubular cells (Fig 4). However, expression of ACE was more diffused in the renal tissue of SCD mice, compared to renal ACE expression in the CON mice, which was more concentrated at the glomeruli (Fig 4).

### Modulation of RAS protein expression and activity in mice with SCD treated with enalapril

**Fig 5:**
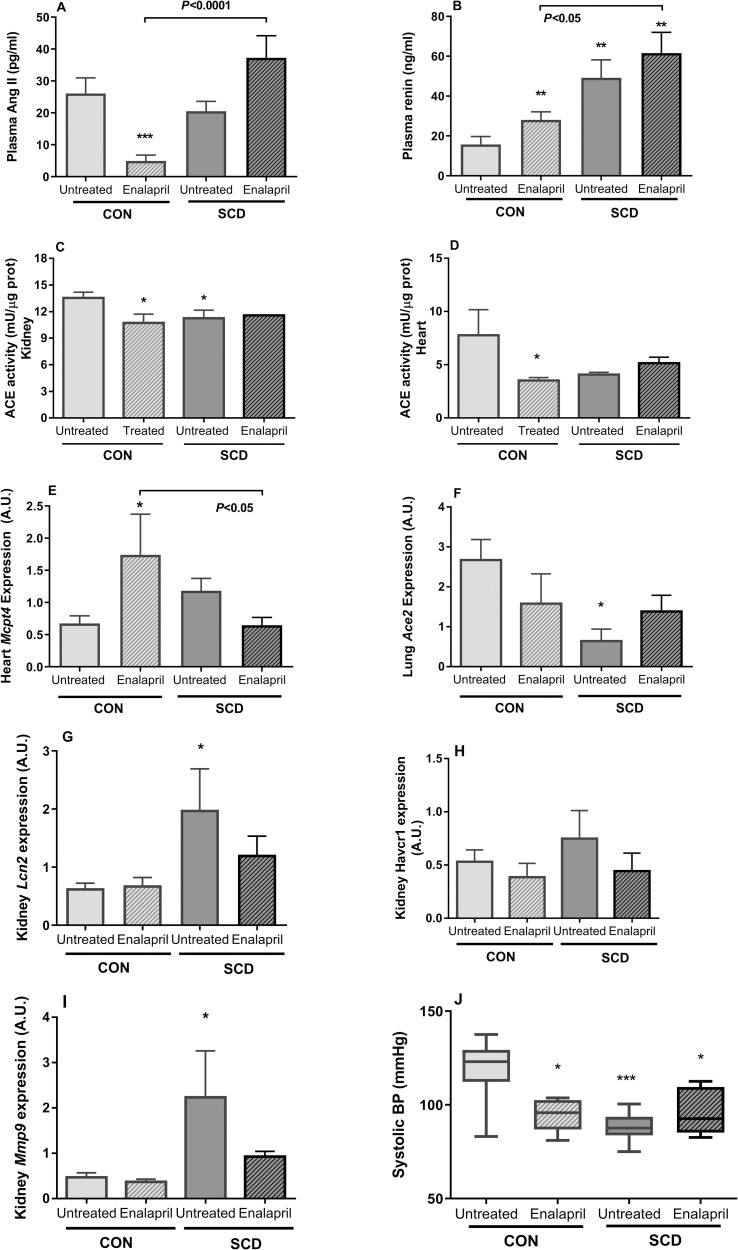
Effects of ACE inhibition upon blood pressure, proteinuria and systemic RAS protein expression and production in SCD mice. Control (CON) and SCD (SCD) mice (5 months old) were treated, or not, for 5 weeks with 25 mg/kg/day enalapril. (A) Plasma Ang II and (B) plasma renin were determined by ELISA at 72 h after the last dose of enalapril (CON, N = 7–11; SCD, N = 7–11); ACE activity in the kidney (C) and heart (D) of mice, as determined by fluorometric assay (CON, N = 3–6; SCD, N = 3–5). Gene expressions of *Mcpt4* (encoding mast cell protease-4) in hearts (E) and *Ace2* (encoding ACE-2) in lungs (F) of mice were determined by qPCR and are depicted as arbitrary units (A.U.) of expression (N = 2–5), relative to the expressions of *Actb* and Gpdh; Gene expressions of (G) *Lcn2* (encoding NGAL), (H) *Havcr1* (encoding KIM-1), and (I) *Mmp9* (encoding MMP9) in the kidneys of mice were determined by qPCR and are depicted as arbitrary units (A.U.) of expression (N = 7–10), relative to the expressions of *Actb* and *Gpdh*; (J) Mean systolic arterial blood pressure (BP); data are means of three measurements on three different days; CON, N = 5–8; SCD, N = 5–8. Values depicted are means ± SEM. *,P<0.05; ***, P<0.001, compared to untreated CON mice.

RAS blocking agents have been employed for treating glomerulopathy in SCD patients; given the potential hypotensive effects of these drugs, we treated chimeric control and SCD mice (5-months old) with 25 mg/kg/day (gavage) enalapril, an ACE inhibitor, for 5 weeks. As expected [31], enalapril significantly reduced plasma Ang II levels in the plasma of control mice, when compared to untreated 5-month old control mice (Fig 5A). In contrast, enalapril did not further reduce plasma Ang II levels in mice with SCD, but in fact, augmented circulating Ang II concentrations to levels that were significantly higher than those of treated CON mice (Fig 5A). Enalapril treatment elevated plasma renin in CON mice (Fig 5B), but did not further elevate plasma renin in SCD mice (Fig 5B).

As anticipated, chronic enalapril administration successfully reduced ACE activity in the kidney (Fig 5C) and heart of CON mice (Fig 5D); however, it did not further inhibit the already lower ACE activities in these organs in SCD mice (Fig 5C and 5D). To investigate a role for ACE-independent Ang II accumulation in SCD mice treated with enalapril, we looked at the murine chymase expression in these mice using qPCR. Expression of the gene encoding mast cell protease-4 (MCP-4; Mcpt4) was elevated by enalapril in the hearts of CON mice, but was suppressed in the hearts of enalapril-treated SCD mice (Fig 5E).

Lungs are a major site of ACE production. The expression of the gene encoding ACE (Ace1) was not significantly modulated by enalapril in the lungs of SCD mice (0.661 ± 0.016 A.U. and 0.581 ± 0.057 A.U. for untreated and enalapril-treated SCD mice, respectively, N = 3, 5). However, the expression of the gene encoding protective ACE-2 (Ace2*)* was lower in the lungs of untreated SCD mice, compared to CON mice, with a non-significant increase in Ace2 expression observed in SCD mice treated with enalapril (Fig 5F). Ace1 expression was not significantly modulated by enalapril in the kidneys of SCD mice (0.340 ± 0.062 A.U. and 0.290 ± 0.032 A.U. for untreated and enalapril-treated SCD mice, respectively, N = 3, 5).

Although clear evidence of a nephroprotective effect of enalapril was not observed, five weeks of 25 mg/kg/day enalapril therapy did not significantly alter *Lcn2* or Mmp9 expression in CON kidneys, but was associated with a non-significant decrease in the renal expressions of these genes in SCD mice (Fig 5H–5J).

### Enalapril administration lowers blood pressure in control mice, but does not exacerbate relative hypotension in mice with SCD

In accordance with the BP regulation effects of enalapril, a significant reduction in systolic BP was observed in healthy CON mice receiving enalapril, when compared with untreated CON mice of the same age (P<0.05, Fig 5J). Importantly, no alteration in mean BP was observed in SCD mice taking enalapril, when compared to untreated SCD mice (P>0.05, Fig 5J).

## Discussion

Renal dysfunction is one of the major complications of SCD and encompasses a variety of pathologies, including tubular defects, leading to urinary concentration deficiency, and glomerular defects resulting in albuminuria. Albuminuria and proteinuria (defined as the presence of proteins in the urine) often develop during childhood in SCD and can progress to chronic kidney disease with advancing age [17, 32]. In addition to age, other factors such as increased hemolysis, anemia, and relative elevation in BP are associated with the onset of kidney disease in SCD. The kidneys are essential for the filtration of blood and excretion of toxins and metabolites via the formation of urine, with the homeostatic balance being maintained by the RAS and Ang II generation [16]. The pathological activation of RAS, with consequent exacerbation in the production of Ang II, can lead to progressive renal damage, as observed in cardiovascular diseases and diabetic and non-diabetic nephropathies [33–36], in association with BP elevation [37].

Consistent with previous observations [6, 9], we found that individuals with SCD displayed lower diastolic BPs than healthy control individuals. However, while anemia is known to increase cardiac output, which can in turn modulate blood pressure and peripheral vasodilation [38, 39], we did not observe any associations between hematological parameters and decrease in BP in SCD. Importantly, mean BP was also reduced in young adult and adult chimeric mice with SCD, indicating that this mouse model represents a reasonable model for the study of cardiovascular alterations and variations in BP in this disease. Significantly decreased serum urea and creatinine were confirmed in the SCA patients studied, as consistently observed in SCA [40–42], and may be indicative of hyperfiltration and impaired urea recovery in the population studied [40, 41]. The decrease in BP observed in the SCD mouse model was associated with increased gene expressions of the NGAL and MMP9 biomarkers in the kidneys of these mice, compared to control mice, indicative of renal injury, specifically tubular damage [43] and glomerular damage [44], respectively; early tubular injury and a progressive loss of renal function have been reported in SCD mice, probably due to continuous processes of ischemia/reperfusion in the organ [45–47].

Given this renal damage and the known association between the RAS and BP regulation, we also measured systemic levels of key proteins of the RAS in humans and mice with SCD. As previously observed [21], plasma levels of Ang II were significantly decreased in older adult chimeric mice with SCD, although another study has reported increased plasma Ang II levels in chimeric SCD [48], but also found increased renal renin expression in these mice. Plasma Ang II was not significantly altered in the population of patients with SCD studied, although such alterations may be more difficult to pick up in humans due to age, genetic modulation and therapies frequently employed for SCD. We did not, however, find any evidence of significant modulation of the Ang II, by hydroxyurea therapy (the current standard of care at most centers) or age, in our patient population. More notably, concentrations of plasma ACE, the enzyme that cleaves Ang I to produce Ang II, were significantly decreased in both humans and mice with SCD, together with reduced circulating enzyme activity of ACE in human SCA individuals and kidney enzyme activity in SCD mice. ACE correlated very significantly with Ang II levels in 5-month old SCD mice and, therefore, the depletion of this enzyme may play some role in modulating Ang II production in SCD. However, increasing evidence demonstrates a key role for renal ACE in BP regulation, where ACE might mediate at least part of its effect through mechanisms independent of Ang I to Ang II conversion and involve other substrates, such as Ang-(1–7) and the potent vasodilator, bradykinin, which is degraded by ACE [49–51]. Trends towards associations between ACE concentrations (but not Ang II) and BP were seen in both human and murine SCD could indicate that other modulators downstream from ACE could be at play in SCD relative hypotension; however, we were unable to evaluate bradykinin or Ang-(1–7) in the current study. The vaso-occlusive processes that occur constantly in the kidney of SCD individuals result in tissue damage and may impair ACE production by the endothelium [52]. Furthermore, the pro-inflammatory cytokines TNF and IL-1β, both elevated in SCD [53], downregulate endothelial ACE expression [54]. Protein expression of ACE was more diffuse in the kidneys of mice with SCD, consistent with our previous report of impaired ACE expression in the kidneys of SCD mice [21], and indicating that reductions in systemic ACE may reflect impaired renal ACE production.

Interestingly, elevated plasma renin was observed in mice with SCD, when compared with control mice of the same age, which may be indicative of the negative feedback effects of Ang II and reductions in afferent arteriolar pressure with subsequent stimulation of renin secretion from the juxtaglomerular cells [55, 56]. Increased plasma renin activity has been reported in pediatric SCA [57] and, indeed ACE inhibitors and angiotensin receptor blockers are known to elevate renin, presumably by interrupting angiotensin II feedback inhibition [55, 58]. It is worth noting that biosynthesis of the steroid hormone, aldosterone, and secretion from the adrenal cortex is controlled by the RAS system and plasma potassium. Aldosterone regulates sodium homeostasis and, therefore, a role for this hormone in the modulation of blood pressure in SCD should not be ruled out. We did not evaluate circulating aldosterone in our SCA patient population, although a recent study reported plasma aldosterone within the normal range in a small group of SCA patients with and without defective tubular acidification [59].

ACEi [22, 48, 60, 61] are often used to treat glomerulopathy in SCD. Roy and colleagues [48] reported that the RAS modulating drugs captopril and losartan reduced albuminuria in mice with SCD, in association with ameliorations in renal pathology. However, authors found that these agents worsened the already impaired urine concentrating ability of these chimeric SCD mice. A recent study in SCD patients in use of ACEi or Ang II receptor blocking agents presented data to suggest that the long-term administration of these drugs was not associated with alterations in systemic BP in these patients [22]. Large studies in humans, however, can be hampered by diversities in patient populations (such as age and clinical factors), and pharmacological studies in mouse models, which are subject to fewer genetic modifying and environmental variations, can provide more reliable data.

Interestingly, while the ability of enalapril administration to diminish ACE activity in the organs of control mice was observed, such an effect was not observed in mice with SCD, possibly due to their already low basal ACE activity. In association with this observation, trends towards increases in plasma Ang II and renin concentrations occurred in SCD mice on enalapril therapy, compared to enalapril-treated CON mice, indicating that the beneficial effects of enalapril in SCD on albuminuria and the prevention of cardiac remodeling [62, 63] may in fact be independent of ACE inhibition. Chymase activity can contribute to ACE- independent Ang II production and accumulation, especially in the heart and blood vessels [64], with chymase-mediated Ang II production implicated in cardiovascular remodeling, rather than BP regulation [65]. MCP-4 is the mouse chymase that displays most similarity to human chymase; while the mouse MCPs can convert Ang I to Ang II [64], MCP-4 can also hydrolyze and inactivate Ang I and Ang II [65, 66], and has pro-inflammatory properties [64]. Whereas MCP-4 gene expression was augmented in the hearts of CON mice following enalapril administration (consistent with previous reports of increased chymase activity in the hearts of ACE-deficient mice [67]), the expression of MCP-4 was suppressed by enalapril in the hearts of SCD mice. While the preliminary nature of these data do not confirm a role for altered chymase activity or chymase-dependent Ang II modulation in murine SCD, it is conceivable that inhibition of cardiac MCP-4 expression could incur benefits in SCD.

## Conclusion

In conclusion, we have confirmed the existence of decreased BP in humans and mice with SCD, in association with decreases in ACE protein concentration and enzyme activity. The benefits conferred by enalapril administration in SCD appear to be independent of ACE activity suppression and Ang II modulation, and may involve the expression and activities of other enzymes, such as ACE-2 and chymases. Future studies should investigate a role for ACE depletion in the mechanisms of BP regulation in SCD and further explore how ACE inhibition approaches improve kidney function, without affecting BP, in SCD.

## Supporting information

S1 Fig Effects of hydroxyurea therapy on blood pressure and plasma RAS proteins in human SCD. (DOCX)

S2 Fig Stratification of blood pressure and plasma RAS proteins by age in human SCD. (DOCX)

S1 Table Primers for quantitative real-time PCR. (DOCX)

S2 Table Correlations between blood pressure, plasma RAS proteins and hematological data in human SCA. (DOCX)

S3 Table Correlations between blood pressure, plasma RAS proteins and circulating markers of renal function in human SCD. (DOCX)

S1 File ARRIVE guidelines checklist. (PDF)
