# Supplementary material for: Reduced blood pressure in sickle cell disease is associated with decreased angiotensin converting enzyme (ACE) activity and is not modulated by ACE inhibition
Source: PLoS One. 2022 Feb 3;17(2):e0263424. doi: 10.1371/journal.pone.0263424 (PMC8812860; doi:10.1371/journal.pone.0263424)
Supplement: S1 Fig — (DOCX) [file pone.0263424.s001.docx]

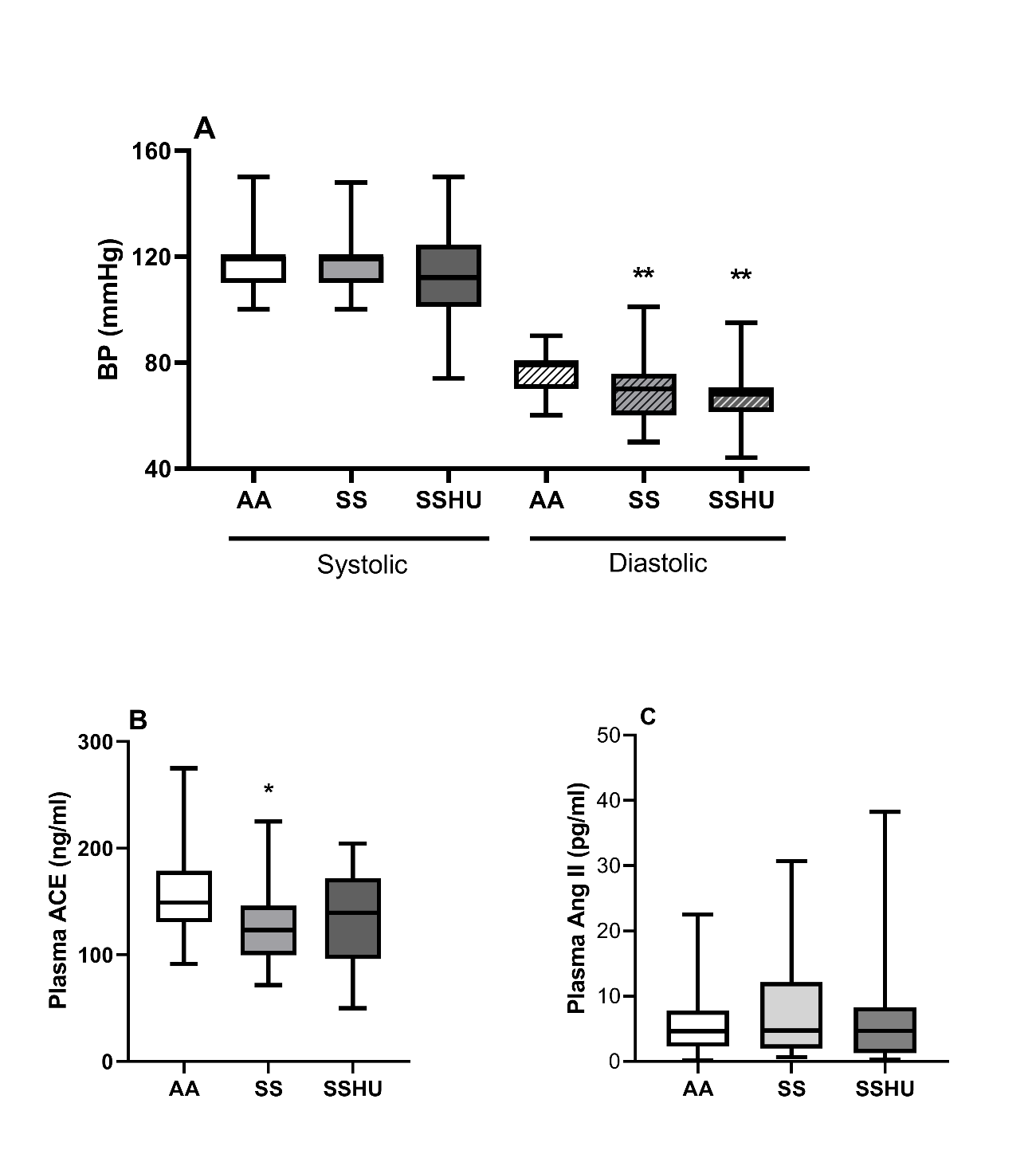


**S1 Figure.** **Effects of hydroxyurea therapy on blood pressure and plasma RAS proteins in human SCD.** (A) Systolic and diastolic blood pressures in healthy control individuals (AA, N=35) and patients with SCA, off (SS, N= 30) and on hydroxyurea therapy (SSHU; N=28; 15-30 mg/kg/day). Plasma ACE (B) and plasma Ang II (C) in patients with SCA off (SS; N=28 and 18, respectively) and on hydroxyurea therapy (SSHU, N=30 and 16, respectively) and healthy individuals (AA, N=40 and 29, respectively). *, P<0.05; **, P<0.01, compared to AA.
