# Supplementary material for: Reduced blood pressure in sickle cell disease is associated with decreased angiotensin converting enzyme (ACE) activity and is not modulated by ACE inhibition
Source: PLoS One. 2022 Feb 3;17(2):e0263424. doi: 10.1371/journal.pone.0263424 (PMC8812860; doi:10.1371/journal.pone.0263424)
Supplement: S2 Fig — (DOCX) [file pone.0263424.s002.docx]

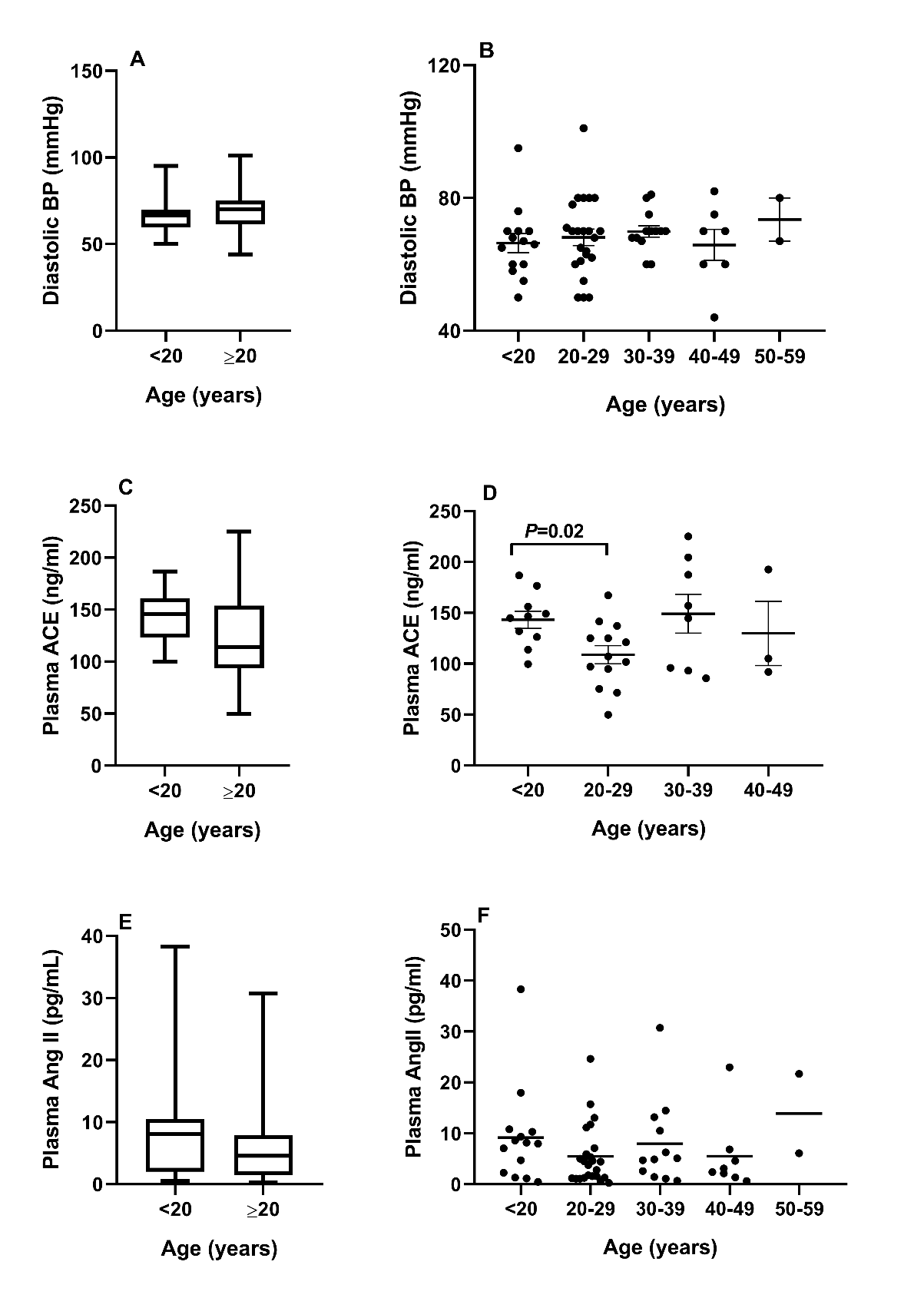


**S2 Figure.** **Stratification of blood pressure and plasma RAS proteins by age in human SCD.** Diastolic blood pressure (A), plasma ACE concentrations (C) and plasma Ang II (E) in patients with SCA, stratified into less than 20 or greater/equal to 20 years of age. Diastolic blood pressure (B), plasma ACE concentrations (D) and plasma Ang II (F) in patients with SCA, stratified into decades of age. ANOVA (non-parametric) and Dunn’s multiple comparisons test.
