# Supplementary material for: Reduced blood pressure in sickle cell disease is associated with decreased angiotensin converting enzyme (ACE) activity and is not modulated by ACE inhibition
Source: PLoS One. 2022 Feb 3;17(2):e0263424. doi: 10.1371/journal.pone.0263424 (PMC8812860; doi:10.1371/journal.pone.0263424)
Supplement: S1 Table — (DOCX) [file pone.0263424.s003.docx]

**S1 Table. Primers for quantitative Real-Time PCR**

| **Gene** | **Protein encoded** | **Primer** | **Optimal primer concentration** |
| --- | --- | --- | --- |
| *Actb* - F  *Actb* - R | β-ACTIN | 5´- ACTGCCGCATCCTCTTCCT -3´  5´- GAACCGCTCGTTGCCAATA- 3´ | 150nM |
| *Gapdh* - F  *Gapdh*-R | GAPDH | 5´- TGCACCACCAACTGCTTA -3´  5´- GGATGCAGGGATGATGTTA -3 | 150nM |
| *Ace* - F  *Ace* - R | ACE | 5′-GGGCATTGACCTAGAGACTGATG-3′  5′-CTTGGGCTGTCCGGTCATAC-3′ | 70nM |
| *Ace2* - F  *Ace2* - R | ACE-2 | 5′-ACCAAAGCATTAAAGTGAGGATAAG-3′ 5′-GTTGTTGGTCCATTCATATGCATT-3′ | 150nM |
| *Havcr1* - F  *Havcr1* - R | KIM-1 | 5′-GCATCTCTAAGCGTGGTTGC-3′  5′-TCAGCTCGGGAATGCACAA-3′. | 150 nM |
| *Lcn2* - F  *Lcn2* - R | LCN2/NGAL | 5′- TGAAGGAACGTTTCACCCGCTTTG -3′  5′- ACAGGAAAGATGGAGTGGCAGACA -3′ | 150 nM |
| *Mcpt4* - F  *Mcpt4* - R | MCPT4 | 5’ – ATTCACAGAGGGAGTCTCTTTG-3´  5’ –GTTCACCCAAAGTACAACTTCTA-3’ | 300nM |
| *Mmp9 -* F  *Mmp9 -* R | MMP9 | 5’ – TGCCCTGGAACTCACACGA -3´  5’ – AACTCACACGCCAGAAGAATT -3´ | 600nM |

 ACE, angiotensin-converting enzyme; GAPDH, glyceraldehyde-3-phosphate dehydrogenase; KIM-1, kidney injury molecule-1; LCN2/NGAL, neutrophil gelatinase-associated lipocalin; MCPT4, murine mast cell protease 4.
