# Supplementary material for: Reduced blood pressure in sickle cell disease is associated with decreased angiotensin converting enzyme (ACE) activity and is not modulated by ACE inhibition
Source: PLoS One. 2022 Feb 3;17(2):e0263424. doi: 10.1371/journal.pone.0263424 (PMC8812860; doi:10.1371/journal.pone.0263424)
Supplement: S2 Table — (DOCX) [file pone.0263424.s004.docx]

**S2 Table. Correlations between blood pressure, plasma RAS proteins and hematological data in human SCA**

|  | Parameter | Fetal Hemoglobin  (%) | Red blood cells (10^6^/µL) | Hemoglobin (g/dL) | Hematocrit  (%) | Reticulocytes (%) | Leukocyte  Count (10^3^/µL) |
| --- | --- | --- | --- | --- | --- | --- | --- |
| Systolic BP | Spearman r  *P* value  N | -0.074  0.617  (N=48) | 0.149  0.226  (N=58) | 0.097  0.463  (N=58) | 0.049  0.712  (N=58) | 0.244  0.081  (N=51) | 0.080  0.538  (N=58) |
| Diastolic BP | Spearman r  *P* value  N | -0.083  0.585  (N=48) | 0.112  0.412  (N=58) | 0.187  0.164  (N=58) | 0.162  0.230  (N=58) | -0.076  0.598  (N=51) | 0.015  0.940  (N=58) |
| Plasma Angiotensin II | Spearman r  *P* value  N | 0.268  0.069  (N=47) | -0.033  0.806  (N=58) | 0.053  0.690  (N=58) | 0.051  0.700  (N=58) | 0.242  0.094  (N=49) | -0.114  0.393  (N=58) |
| Plasma ACE | Spearman r  *P* value  N | -0.037  0.867  (N=23) | 0.038  0.831  (N=34) | 0.050  0.778  (N=34) | 0.028  0.877  (N=34) | -0.111  0.599  (N=34) | 0.007  0.968  (N=34) |

Systolic/diastolic blood pressure (BP, mmHg), plasma Angiotensin II (pg/ml) and plasma ACE (ng/ml), determined in the human SCA patient cohort, were correlated with hematological data using Spearman correlation. Hematological data were obtained using an automated Advia Hematological System (Siemens Healthcare GmbH, Germany). Fetal hemoglobin (HbF) levels were determined using high-performance liquid chromatography (Variant II™, Bio-Rad Laboratories, Hercules, CA, USA).
