# Supplementary material for: Reduced blood pressure in sickle cell disease is associated with decreased angiotensin converting enzyme (ACE) activity and is not modulated by ACE inhibition
Source: PLoS One. 2022 Feb 3;17(2):e0263424. doi: 10.1371/journal.pone.0263424 (PMC8812860; doi:10.1371/journal.pone.0263424)
Supplement: S3 Table — (DOCX) [file pone.0263424.s005.docx]

**S3 Table. Correlations between blood pressure, plasma RAS proteins and circulating markers of renal function in human SCD**

|  | Parameter | Plasma Angiotensin II | Plasma ACE | Urea | Creatinine |
| --- | --- | --- | --- | --- | --- |
| Systolic BP | Spearman r  *P* value  N | \|  \|  \| \| --- \| --- \|   0.077  0.578  (N=54) | 0.033  0.855  (N=31) | -0.280  0.044 *  (N=52) | 0.034  0.811  (N=52) |
| Diastolic BP | Spearman r  *P* value  N | 0.106  0.448  (N=54) | 0.299  0.100  (N=31) | -0.074  0.603  (N=52) | -0.040  0.770  (N=52) |
| Plasma Angiotensin II | Spearman r  *P* value  N |  | -0.051  0.772  (N=32) | -0.024  0.868  (N=50) | -0.068  0.639  (N=50) |
| Plasma ACE | Spearman r  *P* value  N |  |  | 0.223  0.229  (N=31) | -0.125  0.497  (N=31) |

Systolic/diastolic blood pressure (BP, mmHg), plasma Angiotensin II (pg/ml) and plasma ACE (ng/ml) were determined in the human SCA patient cohort (according to Methods section) and were correlated with each other and with blood urea (mg/dL) and creatinine (mg/dL) using Spearman’s correlation; *, P<0.05. Serum urea and creatinine were determined using the AU5800 system (Beckman-Coulter, USA), according to the manufacturer’s protocols.
